# A roadmap for the digital management of long-lasting insecticide-treated mosquito nets (LLINs) distribution campaigns in the Democratic Republic of Congo

**DOI:** 10.11604/pamj.2023.44.195.36755

**Published:** 2023-04-20

**Authors:** Joris Losimba Likwela, Joseph Kalonji Ciula, Albert Kalonji Ntumba, Adrien Nsiala Kumbi, Philippe Lukanu Ngwala

**Affiliations:** 1 Soins de Santé Primaire en Milieu Rural, Association sans but lucratif (SANRU Asbl), Kinshasa, Democratic Republic of Congo,; 2 Department of Public Health, Faculty of Medicine and Pharmacy, University of Kisangani, Kisangani, Democratic Republic of Congo,; 3 Department of Family Medicine and Primary Health Care, Faculty of Medicine, Protestant University in Congo, Kinshasa, Democratic Republic of Congo

**Keywords:** Malaria, digitalization, long-lasting insecticide-treated mosquito nets, mass distribution

## Abstract

The use of the long-lasting insecticide-treated mosquito net (LLIN) is one of the basic interventions recommended by the Global technical strategy for Malaria 2016-2030. Since the start of the LLIN distribution campaigns in 2006 in Democratic Republic of Congo (DRC), it was based on paper tools leading to poor quality data. The first digital campaigns date back to 2014 through “Interchurch medical assistance” (IMA), which used the ODK collect application for recording household count data and LLIN distribution data. In 2020 “Soins de santé primaire en milieu Rurale” (SANRU) developed both household registration and LLIN distribution data recording forms as well as additional modules for supervision, monitoring and training. This article briefly describes the status of the implementation process of this digital-based management of LIIN mass distribution. During the first half of 2020, a roadmap was developed between Sanru and the Global fund to fight Tuberculosis, AIDS and Malaria (GFTAM) on the objectives of digitization, the data to be digitized, and the timelines for implementing the changes. In the last quarter of 2021, an internal Sanru team composed of some members of its technical management, and staff in charge of the digitization of LLIN mass distribution campaign data participated in a document review of the deliverables in comparison with the roadmap and in group discussions. For recording household enumeration data and distribution data, forms configured on smartphones allow data recording and uploading without going through manual calculations and previously necessary transcriptions with management based on paper tools, thus removing sources of errors. Online data delivery and automated production of dashboards allow real-time sharing of information to all stakeholders and shorten data validation times. Feedback to actors in the field is possible thanks to access to information and maps generated on the basis of geolocation data from households. ODK forms for supervision and monitoring have been put in place to ensure that these activities are effectively deployed in the field in accordance with the standards set by geolocating the actors and using the data transmitted online for interactive feedback. The next step is to develop a material flow management module to improve the traceability of inputs.

## Introduction

The use of the long-lasting insecticide-treated mosquito net (LLIN) is one of the basic interventions recommended by the Global Technical Strategy for Malaria 2016-2030 [1]. In DRC, since the start of the LLIN distribution campaigns in 2006 in favor of children under 5, then universal distribution from 2009, their management was based on paper tools allowing the recording of data then their compilation for decision-making. This approach led to poor data quality as a result of summation, compilation, or transcription errors. In addition, the management of the effectivity of the participation of the actors in the various training sessions and their payments constituted enormous challenges to be met. Furthermore, the management of input flow was challenging with delays in the availability of data that did not allow interactive feedback with all stakeholders. The quality of actors´ supervision was also frequently questioned. Over the past two decades, there has been a rapid development of information and communication technologies (ICT) and their application in health [2-4]. Their application in the goals of improving human health has great potential to transform the speed, efficiency, capacity, and impact of health services and programs, including public health. The use of smartphones for data encoding was initiated by “Interchurch medical assistance” (IMA) in a few provinces between 2014 and 2019 and this involved about one in ten campaigns. This approach did not cover training management, supervision management, supply, and inventory management. From 2020, with the Ministry of Health (MOH) and the Against Malaria Foundation (AMF) partnership combined with an incentive from the Malaria portfolio management team for the DRC at the Global Fund to fight Tuberculosis, AIDS and Malaria (GFTAM), the DRC has initiated a process of scaling up digitization for the overall improvement of the management of LLIN distribution campaigns using ICTs, as its beneficial effects in other areas of malaria control and other public health interventions had already been demonstrated elsewhere in Africa [5-7]. This article briefly describes the status of the implementation process of this digital-based management of LIIN mass distribution in the five key areas below; i) Training; ii) registration; iii) distribution; iv) supervision; v) management of purchases and supplies as well as the roadmap for prospective improvement.

## Program evaluation

### Methods

**Figure 1:**
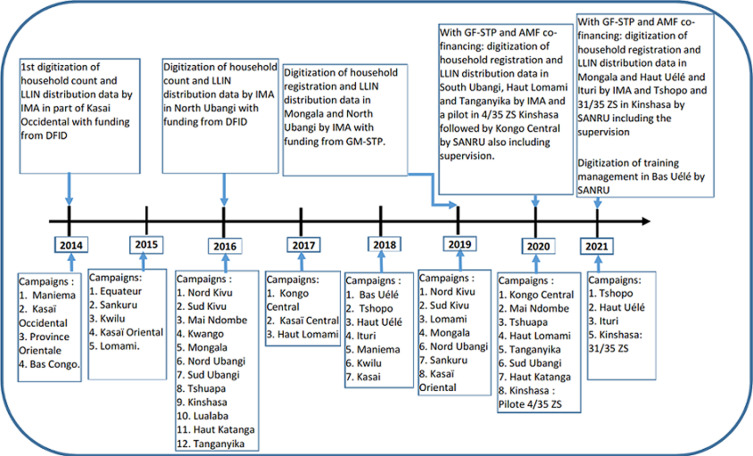
history of the digitalization of the management of long-lasting insecticide-treated mosquito net mass distribution campaigns

**History of digitalization in the management of LLIN mass distribution campaigns in the DRC:** in the DRC, the first digital campaigns date back to 2014 and 2016 through IMA, which used tools based on the ODK collect application for recording household census data and LLIN distribution in parts of the mass distribution campaign of LLINs in the province of Kasai occidental and in the province of North Ubangi using Department for international development (DFID) funding (Figure 1). In 2019, acting as Sanru sub-recipients in the Mongala and North Ubangi provinces, IMA continued to work with similar tools. In 2020 Sanru developed both household registration and LLIN distribution data recording forms as well as additional modules for supervision, monitoring, and training. A materials flow management module is under development.

**Study design:** a descriptive evaluation of the process of digitalization in the management of LLIN mass distribution campaigns was carried out in the period 2018-2021 to summarize the outcomes of the process and discuss challenges and recommendations for future improvement.

**Data collection:** all information was gleaned from content analysis of reports from training workshops, supervision missions, data analysis and validation workshops, and evaluation workshops of LLIN distribution campaigns conducted in the beneficiary provinces during the period. A documentation protocol was instituted at the beginning of the digitization process to standardize the findings in relation to the pre-digitization expectations agreed between Sanru, the National Malaria Control Program (NMCP) and the donors, namely Against malaria foundation (AMF) and the Global fund to fight HIV/AIDS, Tuberculosis and Malaria.

## Results

This section presents the operational details of the implementation of the LLIN distribution campaigns in relation to training, household registration, delivery of LLINs to households, supervision and monitoring, as well as the management of LLIN supplies and inventories prior to digitization, problems to be solved by digitization, and digital solutions implemented.

### Training

**Before digitization:** the training was organized sequentially, going from the provincial level to the Health zone (HZ) level and then to the health area (HA) level. The selection of participants was decentralized and the lists were established with local verification mainly at the level of the HZs. These lists were then used to monitor attendance and ensure payment.

**The problems to be solved through digitization:** the problems to be solved were mainly: i) the difficulties of controlling effective participation (the participants could sign and then leave the room or else ask third parties to sign in their place or could sign after the end of the session) and ii) payment delays relating to the collection and processing of payment lists.

**Digital solutions implemented:** a tool for monitoring of training participation has been developed using two application solutions, one of which is online on the server-side (web application), another local on the client-side. The web application developed in PHP using the postgreSQL database allows to create and manage a training in all its aspects, and to generate a QR code that will be displayed to the local application. The local application designed in C# using the SQLite database can work offline, and is configured by scanning the QR code. It allows users to be registered once and to report their presence each day of the training through their fingerprint. At the end of the training, only the attendance information is sent to the web application which will generate the attendance report and allow the payment of the participants.

### Registration

**Before digitization:** registration agents were assigned a specific number of households to register per day in a geographic area (neighborhood, village, etc.) for a predetermined number of days. For each household, the agent recorded on a voucher and on a copy to be kept: i) the household number; ii) the name of the HZ; iii) the name of the health area; iv) the distribution site; v) the name of the family head; vi) the number of people in the household; vii) the address; viii) the community health worker´s (CHW) name; ix) the registration place and date; x) the signature. The badge number was pre-printed. Then, this same information was transcribed on to a registration sheet. Finally, the agent gave the numbered badge (printed in security ink) to each household and included the information listed above. The agent then retained the copy of the badge with the same information.

**Problems to be solved through digitization:** the problems posed by this management based on paper tools during the registration were, in particular: i) the difficulty of locating households on long paper lists which were sometimes poorly ordered; ii) the fraudulent inflation of the number of households due to the lack of control that the geolocation of households would allow; iii) the difficulty of recovering missed households during the visit by registration agents; iv) the difficulty of supervising hundreds of registration agents without real-time feedback of problems on the ground. After registration, the absence of a database with all the households did not allow: i) the printing of the distribution lists in the desired order (alphabetical, badge number, etc.) to facilitate the distribution; ii) the cross-checking of different parameters among the information collected and with other data sources for analysis; iii) to avoid the deterioration of data quality following summation, compilation or transcription errors and; iv) to avoid delays in data validation following delays in processing and transmission of data from one level to another.

**Digital solutions implemented:** after studying the problems posed, we chose the ODK collect application as a perfectly suited tool to respond by designing forms that are consistent, clear, and sufficiently detailed to significantly reduce errors in using the tool. For this reason, during the design of the forms: formulas were introduced to automatically calculate the number of LLINs to be given to the heads of the household from the number of people declared to reduce the possibility of fraud on the number of LLINs distributed. Constraints were introduced to control user input, options were disabled to prevent the user from freely modifying data after visiting a household, and access to configuration settings was via a conditioned password known only to the independent supervisor based at HZ level to support the HA officer nurse and to support the field teams to resolve any problems in the use of the application in the field. An extraction application was also designed to process, organize, calculate and produce a database in the format corresponding to the Excel database used by the NMCP. It also makes it possible to generate a household map from geolocation data sent by the ODK Collect application installed in the interviewers' smartphones. Raw data can be extracted from different forms: i) registration; ii) rapid monitoring; iii) supervision at the Central Office of HZ (COHZ) and; iv) supervision at the HA level. The registration data is therefore directly available for all parties without manipulation in terms of calculations, or error-prone transcriptions. There is also a time saving which reduces the delay between the end of the registration and the validation of the data at the provincial level. Ultimately, this extraction application generates a dashboard that presents the data collected in a simple format, making it possible to calculate the key indicators for monitoring of household registration, namely: i) number of registration agents per HA, by HZ, by province; ii) number of households registered (possibility of disaggregating by size and type of household) by village/street, by HA, by HZ, by province; iii) number of persons registered (possibility to disaggregate according to relevant age categories for future campaign planning including vaccination or micronutrient supplementation campaigns) by HA, by HZ, by province; iv) an average number of people per household and by HA, by HZ and by province.

### Distribution

**Before digitization:** long-lasting insecticide-treated mosquito net were received at sites based on the number of nets to be distributed at a given site. The community attended the assigned distribution sites with their vouchers to exchange them for the number of mosquito nets corresponding to the size of their household (allocation table defined after registration). The distribution agents then checked the vouchers against the paper lists completed by the registration agents before giving the corresponding number of mosquito nets and checking the name on the list. The distribution agents balanced the nets received, the nets distributed, the remaining stock, and the vouchers received at the end of each day and at the end of the distribution.

**Problems to be solved through digitization:** it took between 2 and 5 minutes for a distribution agent to find the name on the list before distributing the LLINs. This led to long queues. There was no way to verify the identity of the beneficiary other than through possession of the voucher. Households who lost their voucher were asked to return on the last day for checks before the LLINs were handed over without any guarantee that the problem would be resolved. End-of-day inventories and reconciliations were either not always done or not always done correctly. As with the registration, after the distribution, there were delays in validating the data.

**Digital solutions implemented:** the solutions proposed for the registration are the same as those applied for the distribution with ODK forms comprising formulas and input restriction and the extraction application with the functionalities described in the previous section. In the context of COVID-19, the NMCP and its partners having chosen the option of combining the registration with the distribution, the registration and distribution forms have been merged and the smartphone entry and extraction work are carried out simultaneously. The dashboard generated by the extraction application makes it possible to calculate the key indicators for monitoring the distribution of LLINs to the following households: i) number of households served with LLINs (possibility of disaggregating by size and type of households) by health area, by health zone, and by province; ii) number of LLINs distributed (possibility of disaggregating by size and type of households) by HA, by health zone and by province; iii) average number of LLINs per household and this by HA, by health zone and by province.

### Supervision and monitoring

**Before digitization:** supervisors were deployed to the field to ensure that activities were carried out as planned. These supervisors were assigned a supervision area per level: (i) proximity supervisor (IT) for the health area; ii) axis supervisors (members of the HZ team) for 3 HA in rural HZ or 5 HA in urban HZ; iii) zonal supervisor (called local coordinator=head of HZ) for the HZ; iv) provincial supervisor (Member of Provincial health division team) at the rate of one per HZ; v) National supervisor (of the central NMP or the General Secretariat of MOH) at the rate of one for three to four HZ. To ensure campaign quality, monitoring through a visit of a minimum of 10 households per health area per day was carried out by monitoring agents of the principal recipients (PR) of donor funds. The data collected, using ad hoc templates, was presented daily to local coordination committee (LCC) meetings for action.

**Problems to be solved through digitization:** supervisors could not target problem areas during their supervision plan due to a lack of relevant feedback from the field in real-time. It was difficult to control that the supervision was carried out. The monitoring data was not used during the activity due to the lack of timely feedback to the public relation hierarchies for appropriate decision-making.

**Digital solutions implemented:** an ODK Collect form has been developed and configured on smartphones. It includes all the fields provided on the checklist of the supervision template previously used by the Program for the fight against malaria (PNLP) in paper format. It´s completion in the field makes it possible to geo-locate the supervisor and to produce maps making it possible to superimpose the route of the supervisors on those of the field actors (thanks to the geographical coordinates taken when filling out the registration and distribution forms on a smartphone). Thus, it is not only possible to ensure that supervisors can meet with actors in the field, but it also allows each supervisor to obtain support from his supervisor after identifying areas for improvement or other weaknesses based on the registration and distribution data submitted online by the actors in the field. With the extraction application, it is then possible to calculate supervision monitoring indicators such as; i) number of supervisors by HZ and by province and; ii) number of actors supervised by HA, by HZ, and by Province. The online monitoring data are now accessible for use by coordination bodies at the local, provincial and national levels.

### Inventory and supply management

**Before digitization:** transporters contracted by Sanru transported 80% of the mosquito nets from the country entry point to the health zone, with 20% of LLINs stored at the provincial level as a buffer stock while awaiting the registration. After the validation of the micro plans, the remaining 20% were distributed taking into account the confirmed requirements of the HZs with potentially equalization between initial stocks delivered to HZs. Long-lasting insecticide-treated mosquito net received at the warehouses selected by the HZ were kept until the date of distribution was confirmed. After that, the HZ team managed to transport to the HA and sites in a multimodal manner (trucks, cars, bicycles, rickshaws, man's back, motorized or non-motorized canoe, etc.). Stock cards had to be kept in all warehouses (at provincial, health zone, and site level). Shipping slips and receipt slips were countersigned for traceability. At the central and provincial levels, Sanru logisticians transcribed the day-to-day information from the stock cards and the various slips into an Excel database which was then shared at higher levels and then with donors.

**Problems to be solved through digitization:** knowing the statues of each warehouse and where the LLINs are located required multiple communications and confirmation with different actors (carriers, stock managers, etc.). Stock cards were not always properly kept, inventories were not always done and transcription errors were always possible.

**Digital solutions are being implemented:** an online application for the management of stock flow is under development. This application will be based on ODK collect and will capture “static” information from orders to shipment to health zones, AS, and villages and streets, passing through entry gates and provincial warehouses. Dynamic information on stock flow from one point to another as follows: i) record the issue of delivery notes sent by suppliers with quantities, shipping route, time of collection, departure warehouses; ii) record the quantities received at the destination and the time of recording. An online application called “JUPITER” for inventory flow management is currently being developed. This application will be based on two solutions: an ODOO-based solution for the creation of the virtual warehouse network that will capture “static” information and an ODK Collect-based solution for the creation of forms to capture and import “dynamic” information on the flow of LLINs from one point to another, by scanning barcodes, from reception at the gateway warehouses to shipment to health zones, HAs, and villages and streets, via provincial warehouses. The “static” information is about the availability of LLINs in stock at the gateway warehouses, provincial warehouses, HZ warehouses, HA warehouses, and village stores. The dynamic information on the flow of stock from a point to another captured via ODK forms will allow Jupiter to: i) establish delivery bills sent indicating quantities, shipping route, time of collection, departure warehouses; (ii) record quantities received at destination and the time of recording. This static and dynamic information will make it possible to produce a dashboard of the material flow. This dashboard will be used to calculate the following indicators: i) number of LLINs ordered; ii) number of LLINs received/available at a given time at the warehouse at the point of entry into the country; iii) number of destinations for LLINs (provincial warehouse, HZ warehouse, HA storage site, site/village/neighborhood/avenue) by province; iv) number of LLINs moving to different destinations at a given time; v) number of LLINs received/available at a given time at the provincial warehouse level, at the health zone level, at the health center level, at the site/village/neighborhood/avenue level by province; vi) remainder of LLINs at the level of the provincial warehouse, at the level of the HZ, at the level of the HA, at the level of the site/village/district/avenue by province.

## Discussion

Our study is the first in Africa to present not only the benefits of digitizing the management of LLIN distribution campaigns, but also to analyze in detail the implementation challenges to be addressed through this digitization as well as the indicators that can be collected with computerized tools. Aikpon R *et al*. and Likwela JL *et al*. published in 2020 and 2022 on digitization of distribution campaigns in Benin and DRC respectively [7,8]. Aikpon had described the use of biometric data for digital tracking of participants in training sessions using their biometric data to validate their attendance at each training session [7]. He also indicated that a digital money and asset transfer platform was being used to secure the payment of actors and the management of digital tools. Aikpon R *et al*. and Likwela JL *et al*. all emphasized the contribution of the digital tool to the establishment of a robust demographic database of household census and LLIN distribution with the production of an online dashboard, updated in real-time, allowing field supervisors to make important decisions efficiently, and to effectively monitor household coverage rates [7,8]. This data collected on smartphones and available via web-based platforms allowed the identification of missed households with sequential and geospatial analytical dashboards easily accessible to field supervisors. In this paper, we describe the current stage of development of digital tools for managing LLIN distribution campaigns in the DRC. In addition to the aspects of managing effective participation in training and the monitoring of household enumeration and distribution of LLINs to households, we describe the deployment of tools to monitor the effectiveness of supervision and LLIN tracking. For each of these different aspects, we present follow-up indicators that can be used to monitor both the key processes of the campaign and the results. Unlike paper-based tools that only provide aggregated data, data collected with digital tools allow for richer analyzes as individual data are accessible online.

However, all these positive aspects should not overshadow the difficulties of deploying digital tools in the African context, namely: i) low electricity coverage; ii) poor telephone network coverage for data downloading; iii) the profile of some community health workers unsuitable with the ability to use smartphones [8,9]. Indeed, digital tools require information and communication infrastructure, the ability to use that infrastructure, a relatively stable supply of electricity and people to maintain and support the infrastructure [9]. Currently, only 40% of people in Africa use the Internet, and the data-only mobile broadband basket prices as % of gross national income per capita is 5%, 12.5 times higher than in Europe and 3.6 times higher than in Asia-Pacific [10]. Mobile phone penetration is reported to be 64%, a figure which is skewed because penetration is calculated on the number of subscriptions, that is the number of Sim cards in circulation and not the number of people using mobile phones [9]. Africa´s telecommunication infrastructure is poor, in part due to the continent´s long history of civil unrest and war which set economies and infrastructure back by up to seven years for each year of unrest. Rural areas are least likely to be provided because of inadequate infrastructure. In addition, there is limited capacity of digital tools by healthcare workers and community health workers, and lack of governments will [9]. In Africa, a study that gathered the opinions of 30 experts from all over Africa on digital health had noted that some initiatives categorized as “digital health” were national initiatives, but that most of them were at the level of platforms created in the North to serve the countries of the South. According to these experts, this created new threats that would be close to neo-colonialism [11].

The interest of the Sanru Asbl platform is that it is designed from start to finish by national staff of the organization. This response to a call by Mawere and Van Stam for digital solutions developed in situ for sustainable digital health in African societies. Indeed, these authors note that the research and development of digital health necessitates embedding in context. For these authors, the context determines the political components of technology and its relationships, meaning and meaning-making, knowing and ways of knowing [12]. Since the last quarter of last year, the Ministry of Public Health, Hygiene and Prevention (MPHHP) has launched an initiative to capitalize on local experiences in digitizing mass campaigns to build a global platform that can be used for all campaigns (immunization, mass distribution of LLINs, micronutrient supplementation and mass treatments for neglected tropical diseases). Furthermore, the use of personal data, including biometric data, raises issues such as liability, legislation, privacy, data security and consent. Currently, the DRC does not have a law on the use of personal data and biometric data. Thus, personal data are used on site, particularly for monitoring the effectiveness of participation in training and then anonymized before being put online.

## Conclusion

In recent times, the widespread application of information and communications technology (ICT) in the health sector has significantly improved the health care delivery system, such as promoting health care and improving the quality of health interventions [13,14]. Their application in the management of mass LLIN distribution campaigns in the DRC has been a decisive contribution, in particular in the context of COVID-19 where the necessary physical distancing imposes the limitations on training and validation workshops and household registration practices. The NMCP intends to capitalize on the experiences of IMA and Sanru to offer standard campaign digitalization tools for all stakeholders engaged in mass LLIN distribution campaigns in the DRC. Furthermore, the Ministry of Public Health, Hygiene and Prevention (MPHHP) would like to expand this experience to all mass campaigns, including immunization, micronutrient supplementation, and mass treatments for neglected tropical diseases. The NMCP intends to capitalize on the experiences of IMA and Sanru to offer standard campaign digitalization tools for all stakeholders engaged in mass LLIN distribution campaigns in the DRC.
